# Ketamine and its two enantiomers in anesthesiology and psychiatry: A historical review and future directions

**DOI:** 10.1016/j.jatmed.2024.07.001

**Published:** 2024-07-11

**Authors:** Kenji Hashimoto, Mingming Zhao, Tingting Zhu, Xingming Wang, Jianjun Yang

**Affiliations:** a Chiba University Center for Forensic Mental Health, Chiba 260-8670, Japan; b Department of Anesthesiology, Pain and Perioperative Medicine, The First Affiliated Hospital of Zhengzhou University, Zhengzhou 450052, China; c Neuroscience Research Institute, Academy of Medical Sciences, Zhengzhou University, Zhengzhou 450052, China

**Keywords:** Analgesia, Anesthesia, Arketamine, Depression, Esketamine, Ketamine

## Abstract

Ketamine, a dissociative anesthetic, is widely utilized in both human and veterinary anesthesia. Its (*S*)-enantiomer, esketamine, is similarly employed for anesthesia and analgesia. The anesthetic effects of both ketamine and esketamine arise from their antagonism of the *N*-methyl-D-aspartate receptor (NMDAR). In field of psychiatry, the rapid-acting antidepressant properties of ketamine for severe depression have generated significant interest, resulting in its increased off-label usage in the United States (U.S.). In 2019, esketamine nasal spray received approval for use in both the U.S. and Europe. However, concerns have emerged regarding potential adverse effects, including long-term efficacy, addiction risks, and the potential for increased suicide risk in clinical settings. In contrast, arketamine, the (*R*)-enantiomer of ketamine, exhibits superior and longer-lasting antidepressant effects in rodent models of depression, with fewer side effects compared to esketamine. Nevertheless, research on the efficacy and safety of arketamine in patients with depression remains limited. This article provides a concise exploration of the historical use of ketamine and its two enantiomers in anesthesia and psychiatry, while also delving into potential future directions for their application in these fields.

## Introduction

**Fig. 1:**
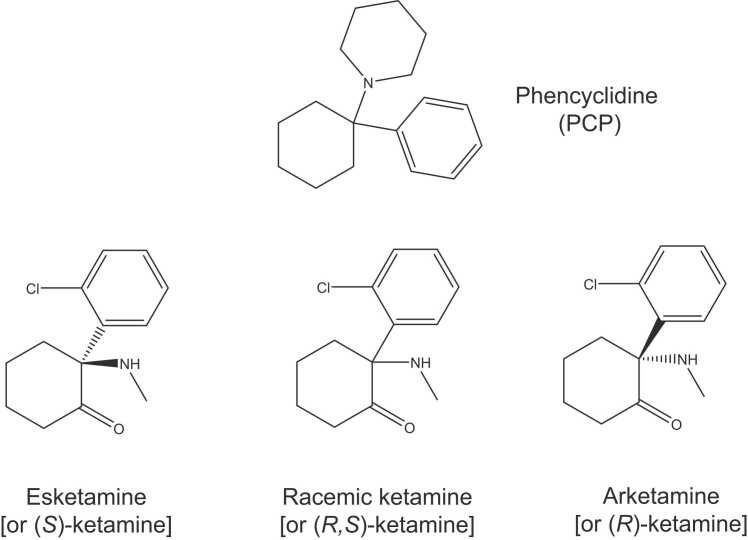
Chemical structure of phencyclidine (PCP), and ketamine, and its two enantiomers esketamine and arketamine.

**Fig. 2:**
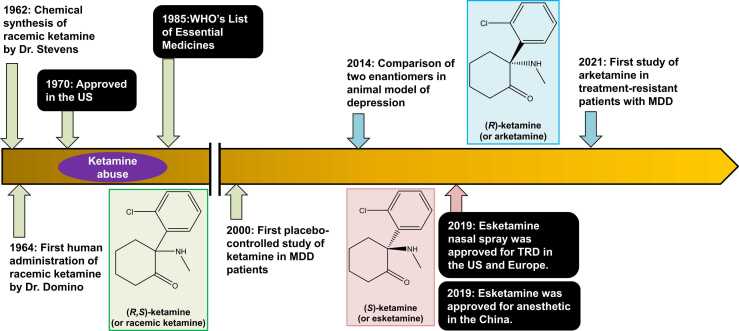
Brief history of ketamine and its two enantiomers in anesthesiology and psychiatry. This figure has been created with minor modifications to the figure in the reference.^6^

Ketamine, also known as (*R,S*)-ketamine (Fig. 1), is extensively utilized as an anesthetic in both human and veterinary medicine due to its distinctive pharmacological properties and safety profile.^1^, ^2^, ^3^, ^4^, ^5^ In human medicine, ketamine is known for inducing a unique "dissociative anesthesia" that preserves protective airway reflexes, supports spontaneous breathing, and maintains stable hemodynamics. These characteristics make it particularly valuable in emergency scenarios or environments lacking advanced airway management equipment. Its minimal impact on cardiovascular stability is noteworthy, often providing sympathetic stimulation, which proves beneficial in states of hypovolemic or shock. Additionally, ketamine is favored for procedural sedation, particularly in pediatric settings, and for painful procedures due to its potent analgesic effects. Its rapid onset and brief duration of action are ideal for shorter procedures. In veterinary applications, ketamine offers similar benefits, providing rapid anesthesia induction with minimal cardiovascular and respiratory disruption. It is used in a wide variety of animals including domestic pets, such as cats and dogs, as well as in wildlife and zoo management. Its versatility is enhanced by its suitability for multiple administration routes, including intravenous, intramuscular, subcutaneous, oral, nasal, and rectal. Recognizing its significance, the World Health Organization (WHO) added ketamine to its Model List of Essential Medicines in 1985 (Fig. 2), acknowledging it as one of the most effective and safe medications vital for health systems.^6^

Ketamine possesses a unique mechanism of action, primarily acting as an antagonist of the *N*-methyl-D-aspartate receptor (NMDAR), which distinguishes it from other anesthetics that typically target γ-aminobutyric acid (GABA) receptors.^7^ This distinctive action facilitates a state of "dissociative anesthesia", where patients achieve analgesia and amnesia without a complete loss of consciousness.^8^ Ketamine comprises two enantiomers: (*S*)-ketamine (or esketamine) and (*R*)-ketamine (or arketamine), each with distinct pharmacological profiles and clinical effects. Esketamine has an affinity for NMDAR that is five times greater than that of arketamine (Table 1), contributing to its anesthetic potency being approximately four times higher.^9^

**Table 1:** Pharmacology of ketamine enantiomers.

Receptors	Binding affinity (Ki)
NMDAR	Esketamine (0.8 μM) > Arketamine (5 μM)
Mu-type of opioid receptors	Esketamine (7 μM) > Arketamine (19 μM)
Kappa-type of opioid receptors	Esketamine (14 μM) > Arketamine (40 μM)
Sigma-1 receptor	Arketamine (27 μM) > Esketamine (131 μM)
Sigma-2 receptor	Arketamine (500 μM) > Esketamine (2800 μM)

Data are from Bonaventura et al.^23^

NMDAR: *N*-methyl-D-aspartate receptor.

In addition to its use in anesthesiology, ketamine has gained significant interest in psychiatry for its rapid and sustained antidepressant effects, especially in treating major depressive disorder (MDD) and treatment-resistant depression (TRD).^10^, ^11^, ^12^, ^13^, ^14^, ^15^ Unlike traditional antidepressants, which may take weeks to exert effects, ketamine can induce significant improvements in depression and suicidal ideation within hours of administration. However, the management of ketamine treatment must be meticulous due to potential side effects, such as dissociation (a sense of detachment from reality), hallucinations, and increased blood pressure. Off-label use of ketamine is prevalent in the United States (U.S.) and Europe.^16^, ^17^, ^18^, ^19^ In an interview with U.S. journalist Don Lemon, Elon Musk, founder of SpaceX and head of the social-media platform X (formerly Twitter), shared his experiences using ketamine to manage a "negative chemical state" akin to depression.^20^ Additionally, the off-label prescription of ketamine by many doctors has seen a significant increase, with prescriptions in the U.S. rising more than fivefold from 2017 to 2022.^20^

This article provides a concise historical review of ketamine and its two enantiomers in both anesthesiology and psychiatry. Additionally, we explore the future prospects of these enantiomers.

## Pharmacology of ketamine enantiomers

Ketamine is generally considered a non-competitive antagonist at the NMDAR, specifically binding to the phencyclidine (PCP: Fig. 1) site in the NMDAR. Additionally, ketamine is known to interact with both opioid and sigma receptors.^7^, ^21^, ^22^ A recent study assessing a panel of 98 receptors and enzymes revealed that at a concentration of 10 μM, both ketamine enantiomers positively bind to the PCP site of the NMDAR (Table 1).^23^ At the same concentration, the mu-type opioid receptor was positively identified for esketamine, but not for arketamine, with no other significant interactions noted at this concentration. According to the data presented in Table 1, esketamine exhibits higher binding affinity (Ki = 7 μM for mu-type and Ki = 14 μM for kappa-type) compared to arketamine (Ki = 19 μM for mu-type and Ki = 40 μM for kappa-type) at opioid receptors.^23^ In contrast, arketamine demonstrates greater affinity (Ki = 27 μM for sigma-1 and Ki = 500 μM for sigma-2) than esketamine (Ki = 131 μM for sigma-1 and Ki = 2800 μM for sigma-2) for sigma receptors (Table 1).^23^ These findings demonstrate distinct pharmacological effects between the two enantiomers.

### Use of ketamine and esketamine in anesthesiology

PCP was first synthesized in the 1950s by the Park-Davis pharmaceutical company and was initially developed as an intravenous anesthetic.^24^, ^25^ Although PCP was marketed in the U.S., its clinical use was curtailed due to severe side effects, including hallucinations, delirium, and psychosis, leading to its discontinuation for medical use in humans by the mid-1960s. Despite being withdrawn from the clinical setting, PCP, also known as angel dust, became a recreational drug noted for its dissociative and hallucinogenic effects.^8^, ^24^, ^25^, ^26^ In pursuit of a safer alternative that maintained the anesthetic benefits of PCP without its debilitating psychological effects, ketamine was synthesized in 1962 by Dr. Calvin Stevens at the Park-Davis pharmaceutical company (Fig. 2). By 1964, Dr. Edward Domino and his colleagues had administered ketamine to human volunteers and confirmed its efficacy for general anesthesia (Fig. 2).^27^ Most participants in this study reported unusual experiences, such as a sensation of floating in outer space and numbness in their arms or legs. Dr. Domino's wife Toni, coined the term "dissociative anesthetic", which is still used today.^8^ Similar to PCP, ketamine can induce hallucination, delirium, and psychosis in humans.^8^, ^24^ However, the LD_50_ of ketamine in rodents is much higher than that of PCP.^28^, ^29^ Therefore, the greater safety of ketamine compared to PCP seemed to be attributed to its higher LD_50_ values.

Ketamine is widely used as a general anesthetic worldwide. Esketamine, known to be four times more potent than arketamine as both an anesthetic and analgesic, is gaining global popularity. In 2019, China approved esketamine as a new anesthetic (Fig. 2), leading to its extensive utilization across the country. Several meta-analyses have shown that perioperative ketamine application reduces post-operative depression and pain scores, although it is associated with an increased risk of adverse effects.^30^, ^31^ Research into esketamine's potential in managing post-operative pain and associated complications such as depression and sleep disturbance is ongoing. A single-center, double-blind, placebo-controlled randomized trial showed that intraoperative esketamine infusion has a prophylactic effect on the incidence of postoperative sleep disturbances in patients undergoing gynecological laparoscopic surgery.^32^ A recent randomized controlled study found that intraoperative esketamine injection during cesarean section prevented the onset of depression at 4 days post-delivery, although this effect did not persist to 42 days.^33^ Conversely, another randomized controlled trial found that while perioperative esketamine administration reduced opioid consumption, it did not decrease the risk of postpartum depression in women following elective cesarean sections.^34^ Despite these mixed results, recent meta-analyses have highlighted the beneficial effects of perioperative esketamine infusion for postpartum depression.^35^, ^36^, ^37^, ^38^

The beneficial effects of esketamine in anesthesia and post-operative care are attracting increasing interest due to its distinct pharmacological properties. In pain management, esketamine significantly enhanced post-operative pain control.^39^, ^40^, ^41^ Its robust analgesic effects, derived from its role as an NMDAR antagonist, are effective in reducing reliance on traditional opioids, which are often linked with higher risks of addiction and other severe side effects. By potentially reducing opioid requirements, esketamine can also decrease opioid-related adverse effects such as respiratory depression, nausea, and constipation.^42^ While esketamine presents benefits in managing post-operative pain and complications, its administration must be carefully monitored due to potential side effects, including dissociative symptoms, elevated blood pressure, and perceptional disturbances (such as hallucinations), which are more prevalent at higher doses. Careful monitoring and dose adjustment are essential to balance efficacy with side effects in a post-operative environment. The higher affinity of esketamine at opioid receptors compared to arketamine may contribute to its effectiveness in pain management (Table 1).^21^, ^23^

Esketamine is used as an anesthetic in children due to its short-acting properties and safety profile.^43^ A recent prospective observational study highlighted the risk of emergence delirium associated with esketamine anesthesia in preschool children following minor surgery.^44^ Therefore, the benefits and drawbacks of using esketamine in pediatric clinical practice should be carefully weighed, particularly when administrating a single-dose in minor surgical procedures.

### Use of ketamine and its two enantiomers in psychiatry

#### Antidepressant effects of ketamine in humans

During the late 1960s and early 1970s, many drugs were used by young people as part of the "make love, not war" protests against the U.S. involvement in Vietnam (Fig. 2).^8^ After the U.S. Food and Drug Administration (FDA) approved ketamine as an anesthetic in 1970, it began to be used recreationally (Fig. 2). At sufficiently high doses, users of ketamine may experience what is known as the "K-hole", characterized by profound dissociation accompanied by visual and auditory hallucinations.^45^ Notably, some drug abusers have sought out ketamine for its rapid-acting antidepressant effects.^8^ Consequently, ketamine has been recognized for its potential rapid-acting antidepressant effects in patients with substance use disorder.^6^

In 2000, Berman and colleagues at Yale University demonstrated that a single intravenous injection of ketamine (0.5 mg/kg) acted within hours to produce rapid antidepressant effects in MDD patients, sustained for up to 72 h post-treatment (Fig. 2).^46^ Numerous subsequent studies confirmed that both single and repeated intravenous infusions of ketamine (0.5 mg/kg) effectively alleviated symptoms of depression and reduced suicidal thoughts in patients with TRD or treatment-resistant bipolar depression.^47^, ^48^, ^49^, ^50^, ^51^, ^52^ Collectively, ketamine has been employed in the treatment of TRD, bipolar depression, and other conditions such as post-traumatic stress disorder, and obsessive-compulsive disorder.^53^, ^54^, ^55^ Recently, the U.S. National Network of Depression Centers issued a position statement advocating for insurance coverage for intravenous ketamine in TRD patients.^56^ Despite regulatory limitations, off-label use of ketamine is popular in the U.S. Importantly, ketamine has rapid antidepressant effects with a favorable safety and tolerability profile, and there is no risk for manic switch. It also appears to act as an anti-anhedonic agent for bipolar depression.^57^ Although long-term data are limited, some researchers suggest that short-term use of ketamine may serve as a disease-modifying strategy.^58^

#### Relationship between ketamine-induced dissociation and antidepressant effects

It has been previously suggested that the dissociative symptoms induced by ketamine injection might be linked to its antidepressant effects in TRD patients.^59^ However, more recent studies^60^, ^61^ have reported that dissociation is not necessary for ketamine’s antidepressant response. Considering the significant role of NMDAR in ketamine-induced dissociation,^62^, ^63^ it appears unlikely that NMDAR plays a major role in the antidepressant actions of ketamine.^61^

#### Nasal spray of esketamine

Given the believed role of NMDAR in the antidepressant effects of ketamine, Janssen pharmaceutical company selected esketamine as a new antidepressant.^64^ A single intravenous injection of esketamine (0.2 and 0.4 mg/kg) produced rapid and sustained antidepressant effects in patients with TRD, although common treatment-emergent adverse events included headache, nausea, and dissociation.^65^ In 2022, Chen and colleagues of Janssen pharmaceutical company^66^ demonstrated that the antidepressant efficacy of esketamine nasal spray in TRD patients showed no correlation with dissociative symptoms. Therefore, these findings suggest that NMDAR may not play a significant role in the antidepressant actions of esketamine.^61^

In 2019, the U.S. FDA and European authorities approved esketamine nasal spray for TRD, despite several concerns about its efficacy and the approval process (Fig. 2).^67^ Esketamine nasal spray has been used in TRD patients alongside current oral antidepressants. This approval was extended to include MDD with acute suicidal ideation and behavior in April 2020. However, a recent analysis from the U.S. FDA Adverse Event Reporting System database has highlighted potential adverse reactions and risks associated with the clinical use of esketamine nasal spray.^68^ These concerns include questions about long-term efficacy, addiction risks, and increased suicidal risks.^68^ Such adverse effects call for careful consideration in the use of esketamine nasal spray.

#### (S)-norketamine as new antidepressant without side effects

Esketamine is metabolized to its major metabolite, (*S*)-norketamine, which has a lower affinity for NMDAR. Similar to esketamine, (*S*)-norketamine has demonstrated rapid antidepressant effects in rodent models of depression, although (*R*)-norketamine did not exhibit antidepressant effects in the same models.^69^ Furthermore, Yokoyama and colleagues reported long-lasting antidepressant effects of (*S*)-norketamine in a social isolation-reared model^70^ and a chronic corticosterone-induced model of depression.^71^ Unlike esketamine, (*S*)-norketamine did not cause behavioral abnormalities, such as deficits in prepulse inhibition, rewarding effects, loss of parvalbumin (PV)-immunoreactivity in the medial prefrontal cortex, or increases in baseline gamma band oscillation.^69^ Given the similarities in their antidepressant effects, esketamine may act as a prodrug for (*S*)-norketamine,^72^ suggesting that NMDAR may not play a major role in antidepressant actions. However, it should be noted that the antidepressant-like effects of (*S*)-norketamine are less potent than those of arketamine.

#### Role of NMDAR inhibition in the antidepressant actions of ketamine

Since NMDARs are believed to play a significant role in the antidepressant effects of ketamine, clinical trials have tested a number of NMDAR-related compounds. Unfortunately, none of these compounds have replicated the robust antidepressant effects of ketamine in MDD patients.^73^, ^74^ Notably, the highly selective NMDAR antagonist (+)-MK-801, also known as dizocilpine, did not produce antidepressant effects in MDD patients [unpublished data by Merck]. Two meta-analyses have shown that intravenous ketamine appears to be more efficacious than intranasal esketamine in treating depression.^75^, ^76^ An analysis of three studies^77^, ^78^, ^79^ comparing intravenous ketamine and intranasal esketamine found similar efficacy for TRD, but intravenous ketamine elicited a faster response.^80^ The bioavailability of intranasal esketamine in humans is known to be lower than that of intravenous ketamine.^10^ However, further studies are crucial to investigate the efficacy and safety of both intravenous ketamine (or arketamine) and intravenous esketamine in TRD patients.

#### Arketamine as new antidepressant without side effects in rodents and monkeys

In 2014, Zhang and colleagues reported that arketamine exhibited greater potency and longer-lasting antidepressant-like effects than esketamine in a neonatal dexamethasone exposure model of depression (Fig. 2).^81^ Subsequent studies confirmed that arketamine has greater and longer-lasting antidepressant-like effects compared to esketamine in various models of depression, including chronic social defeat stress (CSDS), learned helplessness, and lipopolysaccharide (LPS)-treated mice.^82^, ^83^, ^84^, ^85^, ^86^ Collectively, arketamine would be a new antidepressant without side effects.^10^, ^87^, ^88^, ^89^, ^90^ The superior efficacy of arketamine over esketamine in rodent models was also validated by other research groups.^13^

Ketamine is known to cause several behavioral abnormalities in humans, such as psychotomimetic effects, dissociation, and potential for abuse. Preclinical studies in rodents have shown that arketamine induces fewer behavioral abnormalities such as hyperlocomotion, prepulse inhibition, and abuse liability compared to esketamine.^23^, ^82^ Regarding abuse potentail, it is suggested that the abuse liability of ketamine in humans primarily stems from the pharmacological effects of esketamine, particularly NMDAR inhibition and mu-type of opioid receptor activation, rather than arketamine.^23^, ^91^ Additionally, repeated intermittent administration of esketamine, but not arketamine, led to a reduction in PV-immunoreactivity in the prefrontal cortex^92^ and an increase in locomotion following methamphetamine administration.^93^ In a study using conscious monkeys and positron emission tomography, a single intravenous injection of esketamine (0.5 mg/kg), but not arketamine (0.5 mg/kg), decreased the binding availability of dopamine D_2/3_ receptor in the monkey striatum,^94^ indicating that arketamine does not trigger dopamine release in the striatum. Collectively, these preclinical studies suggest that arketamine has fewer side effects than esketamine.

#### Arketamine in humans

Currently, there are few articles documenting the effects of arketamine in humans. In 1997, Vollenweider and colleagues^95^ reported that psychotomimetic doses of esketamine significantly increased the cerebral metabolic rates of glucose in the frontal cortex of healthy volunteers. These metabolic changes were associated with ego-disintegration and hallucinatory phenomena. In contrast, equimolar doses of arketamine tended to decrease glucose metabolism across brain regions. Notably, arketamine did not induce psychotic symptoms; instead, it elicited a state of relaxation.^95^

There are relatively few studies on the antidepressant effects of arketamine in TRD patients. An open-label pilot study in Brazil showed that a single intravenous infusion of arketamine (0.5 mg/kg) produced rapid and sustained antidepressant effects in a small group of female TRD patients (n = 7).^96^ However, a subsequent placebo-controlled pilot study by the same team found that arketamine did not significantly outperform a placebo in TRD patients (n = 10).^97^ Additionally, this research team reported that intravenous infusions of arketamine (0.5 and 1.0 mg/kg) demonstrated rapid-acting antidepressant effects in a small cohort of patients with bipolar depression (n = 6).^98^ Across these three studies, the reported side effects of arketamine, such as dissociation, were very low.^96^, ^97^, ^98^

In January 2023, a press release from Perception Neuroscience (New York, U.S.) stated that the phase 2a trial (NCT05414422) of PCN-101 (arketamine) in TRD patients did not achieve statistical significance on the primary endpoint.^99^ However, further analysis from the phase 2a trial data showed differences between the U.S. and European cohorts. Notably, the U.S. subgroup exhibited clinically meaningful improvements in depression scores for up to two weeks following a single intravenous infusion of arketamine (60 mg). Arketamine was generally well-tolerated in this trial, with no serious adverse events and an acceptable safety profile. There were no significant differences in sedation and dissociative symptoms between the arketamine and placebo groups. Case analyses among the trial (NCT05414422) revealed significant symptom reduction and improvements in social and vocational functioning, including reduced sick leaves and hospitalization post-treatment.^100^ Overall, these findings suggest that arketamine does not produce dissociative side effects in humans at doses effective for treating depression.^61^ Despite the small sample sizes, future double-blind, randomized controlled trials with larger sample sizes are necessary to confirm the efficacy and safety of arketamine in treating depression.

### Mechanisms of action of ketamine and esketamine in anesthesiology

**Fig. 3:**
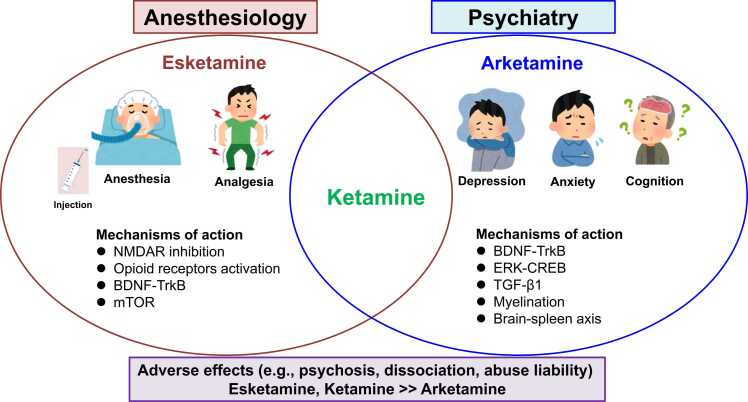
Summary of the mechanisms of action for ketamine enantiomers in anesthesiology and psychiatry. This figure was created based on data using two enantiomers. Portions of the figure was designed using resources from www.irasutoya.com (accessed on 15 July 2024).

Ketamine and esketamine are widely used as anesthetics due to their unique mechanisms of action. Their primary anesthetic and analgesic effects stem from antagonizing the NMDAR in the brain (Fig. 3). Besides NMDAR, ketamine and esketamine influence other neurotransmitter systems, including opioid receptors and monoaminergic receptors (dopamine, serotonin, and norepinephrine), which may contribute to their sedative and euphoric effects (Table 1). Although opioids are generally more potent than ketamine or esketamine for managing acute and straightforward pain scenarios,^101^ a systematic review indicated that ketamine reduces pain more effectively than opioids and causes less nausea and vomiting, albeit with a higher risk of agitation.^102^ Due to the high addictive potential of opioids, their overprescription has been a significant factor in the increase of overdose deaths in the U.S.^103^ Consequently, ketamine and esketamine offer valuable alternatives or adjuncts in situations where opioids are less effective, carry higher risks, or when pain involves a significant neuropathic component.

Chronic pain is often associated with diminished working memory. In 2024, Jiang and colleagues^104^ reported that multiple doses of esketamine improved working memory deficits in a chronic constriction injury mouse model without affecting mechanical or thermal pain sensitivity. This study suggests roles of brain-derived neurotrophic factor (BDNF), its receptor TrkB system, and the gut-brain axis in the beneficial effects of esketamine on working memory deficits in this model.^104^ Additionally, Tan and colleagues found that cognitive deficits induced by PCP in mice could be ameliorated following repeated administration of arketamine, but not esketamine.^105^ Moreover, administering arketamine intermittently during the juvenile and adolescent stages prevented the onset of cognitive deficits in offspring following maternal immune activation,^106^ indicating that arketamine could potentially serve as a prophylactic medication for young individuals at high-risk for psychosis. Consequently, arketamine could potentially be a therapeutic drug for cognitive impairments in humans.^13^, ^107^

### Mechanisms of action of ketamine and its enantiomers in psychiatry

The molecular and cellular mechanisms underlying the rapid and sustained antidepressant actions of ketamine are complex. We have previously published several review articles focusing on these mechanisms.^10^, ^11^, ^12^, ^13^, ^108^, ^109^, ^110^, ^111^ Additionally, other research groups have also contributed review articles on ketamine’s antidepressant actions.^14^, ^15^, ^112^, ^113^, ^114^ In this section, we present updated findings on the antidepressant effects of ketamine and its enantiomers in rodent models.

#### Neurotrophic and growth factors

It is widely recognized that BDNF-TrkB signaling is crucial for the antidepressant effects of ketamine and its enantiomers (Fig. 3).^82^, ^85^, ^115^, ^116^, ^117^, ^118^ A study utilizing both enantiomers revealed that the mechanistic target of rapamycin (mTOR) signaling contributes to the antidepressant effects of esketamine, while extracellular signal-regulated kinase (ERK) may be involved in arketamine's antidepressant effects in a mouse model of depression (Fig. 3).^85^ In 2021, Qu et al.^119^ reported that arketamine could improve depression-like behaviors in *Nrf2* KO mice through BDNF-TrkB signaling. In the following year, Yao and colleagues identified a novel pathway involving ERK-NRBP1 (nuclear receptor-binding protein 1)-CREB (cAMP response element binding protein)- BDNF in microglia, which mediates the antidepressant action of arketamine in a CSDS model (Fig. 3).^120^ Additionally, using RNA-sequencing analysis, Zhang and colleagues discovered that transforming growth factor β-1 (TGF-β1) might contribute to the differing antidepressant effects of arketamine and esketamine in a CSDS model,^121^ and that TGF-β1 could elicit rapid-acting antidepressant effects.^121^, ^122^ Intriguingly, the TrkB antagonist ANA-12 blocked the antidepressant effects of TGF-β1 in a CSDS model.^122^ Additionally, a recent study demonstrated that the oxidative phosphorylation pathway and TGF-β1 play significant roles in the antidepressant effects of arketamine in a CSDS model. These effects are mediated through the spleen-brain axis via the vagus nerve.^123^ Collectively, it is likely that the spleen-brain axis may contribute to the antidepressant effects of arketamine, although further research is needed.^13^, ^90^, ^123^

Furthermore, Deyama and colleagues reported that vascular endothelial growth factor (VEGF) and insulin-like growth factor 1 (IGF-1) in the medial prefrontal cortex play a role in the antidepressant effects of ketamine.^124^, ^125^, ^126^ However, it remains unclear which enantiomer is responsible for these effects. Collectively, these findings suggest that growth factors such as TGF-β1, VEGF, IGF-1 could contribute to the antidepressant-like effects of ketamine and arketamine through BDNF-TrkB activation (Fig. 3).

#### GABA systems

Alterations in glutamatergic and GABAergic neurotransmission play a role in depression.^63^, ^127^, ^128^ In 2023, Tang et al.^129^ reported that ketamine increased GABA levels and decreased glutamate levels in the hippocampus of mice subjected to forced swimming stress.^129^ They observed a significant correlation between GABA levels and depression-like behavior. Additionally, ketamine enhanced the levels of enzymes and transporters on the GABAergic neurons and astrocytes, without affecting those in glutamatergic neurons. It also led to decreased expression of GABA_A_R α1 subunit, increased GABA synthesis and metabolism in GABAergic neurons, change in astrocytic plasticity, and higher ATP (adenosine triphosphate) contents. Remarkably, the GABA_A_R antagonist bicuculline or ATP administration produced rapid antidepressant-like effects, whereas pretreatment with GABA_A_R agonist muscimol blocked the antidepressant-like effects of ketamine, suggesting that ketamine's rapid antidepressant-like action may involve increased GABA synthesis and astrocyte plasticity through the downregulation of GABA_A_R α1 and conversion of GABA into ATP.^129^

#### Anti-inflammatory actions

Inflammation is well documented to play a role in depression.^130^, ^131^, ^132^ Unlike the hallucinogenic psychedelic DOI, arketamine is reported to produce rapid-acting antidepressant effects in mice treated with LPS.^133^ CD38 (cluster of differentiation 38), known for its role in immune responses and cell proliferation, which contributes to inflammation-related psychiatric disorders. Zhang and colleagues demonstrated that the antidepressant effects of arketamine are linked to its anti-inflammatory properties by inhibiting increased CD38 expression in the hippocampus and the reversal of synaptic defects in LPS-treated mice.^134^ Collectively, these findings indicate that arketamine has potent anti-inflammatory effects, which lead to rapid-acting antidepressant outcomes in inflammation-related depression.

#### Brain regions for antidepressant actions of arketamine

Using a learned helplessness model, Shirayama et al.^135^ showed that a single bilateral infusion of arketamine into the infralimbic portion of medial prefrontal cortex, CA3, and dentate gyrus of the hippocampus exhibited antidepressant effects. In contrast, a single infusion into prelimbic portion of medial prefrontal cortex, shell and core of nucleus accumbens, basolateral amygdala and central nucleus of the amygdala, had no effect. Collectively, it is likely that medial prefrontal cortex and hippocampus play a role in antidepressant-like effects of arketamine in rodents.^135^

In 2024, Yokoyama and colleagues^136^ identified a unique activation pattern in the anterior insular cortex (aIC) of social isolated mice treated with arketamine, distinct from other brain regions. Fiber photometry recording from freely moving mice indicated that social isolation reduced aIC neuronal activation upon social contact, and that arketamine, but not esketamine, reversed this reduction. Furthermore, arketamine enhanced social cognition in socially isolated mice during a social memory test, and inactivating the aIC negated the beneficial effects of arketamine on social memory. These findings suggest that arketamine could be an effective intervention for social cognitive deficits by restoring aIC function.^136^

#### Myelination

Myelination and oligodendrocyte functions play a role in psychiatric disorders such as depression and cognition.^137^, ^138^ Using spatial transcriptomics, Huang and colleagues explored the mechanisms behind ketamine’s lasting effects in the medial prefrontal cortex and hippocampus of mice subjected to CSDS, identifying several differentially expressed myelin-related genes.^139^ They demonstrated that ketamine promotes the differentiation of oligodendrocyte precursor cells into mature oligodendrocytes, thereby restoring impaired myelination. Additionally, inhibiting the expression of myelin-associated oligodendrocytic basic protein blocked the long-lasting antidepressant effects of ketamine. Notably, arketamine had a more pronounced effect on myelination than esketamine, potentially explaining its longer-lasting antidepressant effects. These findings reveal novel mechanisms underlying ketamine's sustained antidepressant properties and highlight differences between the two enantiomers.^139^ Furthermore, Wang et al. demonstrated that arketamine could alleviate demyelination and the progression of experimental autoimmune encephalomyelitis^140^ and the demyelination in the brains of cuprizone-treated mice.^141^ Collectively, these studies suggest that arketamine might be a potential therapeutic drug for demyelinating diseases such as multiple sclerosis and depression (Fig. 3).^108^, ^139^, ^140^

#### Opioid receptors

As previously mentioned, ketamine and esketamine are recognized for their binding to opioid receptor subtypes (Table 1 **and** Fig. 3), which contribute to their pharmacological effects. In 2018, Williams and colleagues reported that pretreatment with the opioid receptor antagonist naloxone blocked antidepressant effects in TRD patients (n = 12), albeit it did not affect ketamine-induced dissociation.^142^ This study, based on a small sample size, suggest that the acute antidepressant effects of ketamine require opioid receptor activation. Conversely, opioid receptor activation does not seem necessary for antidepressant effects in patients with depression and alcohol use disorder.^143^ Due to the limited sample size and reliance on case reports, further comprehensive clinical studies with larger sample sizes will be necessary to validate the hypothesis that the opioid system contributes to the antidepressant effects of ketamine (or esketamine) in patients with depression.

In a preclinical study, Zhang et al.^144^ reported that pretreatment with naloxone did not prevent the antidepressant-like effects of ketamine in the CSDS model and LPS-treated inflammation model, indicating that the opioid system may not be involved in the antidepressant actions of ketamine. In contrast, Klein et al.^145^ demonstrated that an opioid antagonist abolished the antidepressant effects of ketamine in rat model, although activation of opioid receptors alone is not sufficient to produce ketamine-like antidepressant effects. Furthermore, pretreatment with the opioid antagonist naltrexone significantly blocked the antidepressant effects of ketamine in rats, while ketamine rapidly increased levels of β-endorphin and the expression of the mu-type opioid receptor gene (*Oprm1*) in the medial prefrontal cortex, along with the expression of proopiomelanocortin, the precursor of β-endorphin, in the hypothalamus.^146^ This study suggests that the increase in β-endorphin and subsequent activation of opioid receptors may contribute to the antidepressant effects of ketamine.^146^ However, it remains unclear which enantiomers can stimulate the release of β-endorphin in the antidepressant effects of ketamine. Additionally, it is uncertain whether NMDAR inhibition can contribute to the release of β-endorphin in the antidepressant effects of ketamine. Further studies using the selective NMDAR antagonist dizocilpine are warranted. Detailed research involving both enantiomers of ketamine is expected to determine the roles of NMDAR and opioid receptors in the antidepressant effects of ketamine and its enantiomers.^147^

#### Sigma-1 receptors

Arketamine has been shown to be more potent at sigma-1 receptor than esketamine, which aligns with the relative antidepressant efficacy of these two enantiomers in rodents (Table 1). Considering the involvement of the sigma-1 receptor in various psychiatric disorders, including depression,^22^, ^148^, ^149^, ^150^ it appears that this receptor may contribute to the distinct effects of the ketamine enantiomers. However, it is important to note that fluvoxamine, a selective serotonin reuptake inhibitor with potent sigma-1 receptor agonism, does not exhibit ketamine-like rapid-acting antidepressant effects in patients with MDD. Interestingly, the classic psychedelic *N,N*-dimethyltryptamine (DMT) is proposed to be an endogenous regulator of the sigma-1 receptor.^151^ Given the growing interest in classic psychedelics (e.g., DMT) for treating psychiatric disorders,^61^, ^152^, ^153^, ^154^ further investigation into the role of sigma-1 receptor in the antidepressant actions of ketamine and its enantiomers is justified.

#### Gut-brain axis

Emerging evidence highlights the crucial role of the gut-brain axis in various psychiatric disorders, including depression.^150^, ^155^, ^156^, ^157^ Wilkowska et al.^158^ suggest that gut microbiota may partially modulate the antidepressant-like effects of ketamine in rodents. Preclinical studies suggest that the gut-brain axis may underlie some of the beneficial effects of ketamine and arketamine in stress models of depression,^159^, ^160^, ^161^, ^162^, ^163^, ^164^ as well as in ovariectomized mice^165^ and cuprizone-treated mice.^141^ Collectively, these findings indicate that the gut-brain axis could play a significant role in the therapeutic effects of arketamine across several disease models, although further detailed studies are needed to clarify this relationship.^10^, ^13^, ^166^

#### Long-lasting prophylactic effects of arketamine

Ketamine is well known for its long-lasting prophylactic effects in stress-exposed mice and LPS-treated mice, despite its short half-life.^167^ Ma and colleagues have demonstrated that the nuclear factor of activated cells 4 (NFATc4) in the prefrontal cortex^168^ and the splenic heme biosynthesis pathway^169^ may contribute to the prophylactic effects of arketamine in LPS-treated mice. Additionally, Ma et al. revealed that microRNA-132-5p and microRNA-149 in the prefrontal cortex are involved in the prophylactic effects of arketamine in LPS-treated mice and in mice subjected to chronic restrain stress, respectively.^170^, ^171^ Collectively, these findings highlight several new signaling pathways and microRNAs that may contribute to the long-lasting prophylactic effects of arketamine, suggesting its potential to prevent relapse in patients with recurrent MDD.

## Conclusion and future directions

Ketamine and esketamine, used as anesthetics, primarily exert their effects through NMDAR antagonism, contributing to their distinct dissociative anesthesia and analgesic properties (Fig. 3). A recent review indicated that ketamine and esketamine are not "the same" at clinically equivalent analgesic and anesthetic dose: psychotomimetic effects are mainly related to NMDAR blockade, and esketamine is not free from unwanted mental impacts.^172^ Future directions for these compounds in anesthesia are likely to focus on enhanced safety profiles, targeted applications, combination therapies, and personalized medicine, potentially increasing their value in anesthesiology.

As discussed in the article, the mechanisms underlying the antidepressant and other beneficial effects of ketamine and its enantiomers are complex. There is growing interest in the two enantiomers in psychiatry.^173^, ^174^, ^175^, ^176^, ^177^, ^178^, ^179^ Preclinical data suggest that arketamine may have more substantial and longer-lasting antidepressant effects than ketamine and esketamine, with fewer adverse effects due to its lower affinity for NMDAR (Fig. 3). However, studies on the efficacy and safety of arketamine in patients with MDD or TRD are currently limited. Further research with larger sample sizes is crucial to determine which enantiomers could deliver robust antidepressant effects of ketamine in patients with depression and other psychiatric disorders. If arketamine can provide rapid-acting and sustained antidepressant effects in depressed patients, it could represent a novel antidepressant option without the side effects associated with ketamine.

Finally, preclinical studies have shown that the antidepressant-like effects of (*S*)-norketamine in rodents are similar to those of esketamine, but with fewer side effects.^69^ This suggests that esketamine for depression may act as a prodrug for (*S*)-norketamine.^72^ Given the adverse effects of esketamine in humans,^68^ (*S*)-norketamine could potentially be a new therapeutic drug without these side effects. Therefore, conducting a randomized, double-blind, placebo-controlled study of (*S*)-norketamine in patients with MDD is of great interest.

## CRediT authorship contribution statement

**Kenji Hashimoto:** Writing – review & editing, Writing – original draft, Supervision, Investigation, Conceptualization. **Mingming Zhao:** Writing – review & editing, Investigation, Conceptualization. **Tingting Zhu:** Writing – review & editing, Investigation, Conceptualization. **Xingming Wang:** Writing – review & editing, Investigation, Conceptualization. **Jianjun Yang:** Writing – review & editing, Supervision, Investigation, Funding acquisition, Conceptualization.

## Disclosure statement

Not applicable.

## Ethical statement

Not applicable.

## Funding

This study was supported by the grant from the 10.13039/501100001809National Natural Science Foundation of China (No. U23A20421 to JJY).

## Declaration of competing interest

Kenji Hashimoto is an editorial board member of Journal of Anesthesia and Translational Medicine. Kenji Hashimoto is the inventor of filed patent applications on "The use of *R*-ketamine in the treatment of psychiatric diseases", "(*S*)-norketamine and salt thereof as pharmaceutical", "*R*-ketamine and derivative thereof as prophylactic or therapeutic agent for neurodegeneration disease or recognition function disorder", "Preventive or therapeutic agent and pharmaceutical composition for inflammatory diseases or bone diseases", "*R*-ketamine and its derivatives as a preventive or therapeutic agent for a neurodevelopmental disorder", and "TGF-β1 in the treatment of depression" by the Chiba University. Kenji Hashimoto also declares that he has received research support and consultant from Otsuka, and Perception Neuroscience. Jianjun Yang is an Editor-in-Chief of Journal of Anesthesia and Translational Medicine. Kenji Hashimoto and Jianjun Yang were blinded from reviewing or making decisions for the manuscript. The authors declare that they have no conflicts of interest.

## Data Availability

None.
